# Hybrid biofunctional nanostructures as stimuli-responsive catalytic systems

**DOI:** 10.3762/bjoc.6.98

**Published:** 2010-09-16

**Authors:** Gernot U Marten, Thorsten Gelbrich, Annette M Schmidt

**Affiliations:** 1 Institut für Organische Chemie und Makromolekulare Chemie, Heinrich-Heine-Universität Düsseldorf, Universitätsstr. 1, D-40225 Düsseldorf, Germany; 2 Department of Chemistry, Universität zu Köln, Luxemburger Str. 116, D-50923 Köln, Germany; 3 present address: Qiagen GmbH Hilden, Germany

**Keywords:** biocatalysis, biolabelling, core–shell nanoparticles, immobilization matrix, thermoflocculation

## Abstract

A novel active biocatalytic reaction system is proposed by covalently immobilizing porcine pancreas trypsin within the thermoresponsive polymer shell of superparamagnetic Fe_3_O_4_ nanoparticles.

Active ester-functional nanocarriers suitable for the immobilization of amino functional targets are obtained in a single polymerization step by grafting-from copolymerization of an active ester monomer from superparamagnetic cores. The comonomer, oligo(ethylene glycol) methyl ether methacrylate, has excellent water solubility at room temperature, biocompatibility, and a tunable lower critical solution temperature (LCST) in water. The phase separation can alternatively be initiated by magnetic heating caused by magnetic losses in ac magnetic fields.

The immobilization of porcine pancreas trypsin to the core–shell nanoparticles results in highly active, nanoparticulate biocatalysts that can easily be separated magnetically. The enzymatic activity of the obtained biocatalyst system can be influenced by outer stimuli, such as temperature and external magnetic fields, by utilizing the LCST of the copolymer shell.

## Introduction

The use of an external stimulus for the activation of (bio)chemical reaction processes can be a valuable tool in fundamental research and in applications such as reaction kits or lab-on-a-chip systems, as it allows for on-demand triggering of an active catalyst, and ideally can be limited, if necessary, to a geometrical confinement. By controlling the catalytic activity of a particulate carrier by a switchable stimulus, the reaction rate of the catalysed process can be significantly accelerated or slowed down. One promising stimulus in this respect is the change of temperature. Polymeric materials show a wide range of thermoresponsive effects that can be explored for a discontinuous change of diffusion or reaction rate [1–3]. The temperature increase can for example be restricted locally by using near infrared (NIR) irradiation [4]. Other systems use a photodynamic process to force a medical effect by the photochemical drug release during light irradiation [5].

By combining thermoresponsive polymers with magnetic nanoparticles, hybrid materials become accessible that can be manipulated by two different stimuli, temperature and magnetic fields. Such dual responsive materials are of interest for a variety of applications ranging from magnetic separation or drug release systems to sensors and actuation [6–13]. We, and other groups, have demonstrated that magnetite nanoparticles decorated with a stabilizing shell composed of LCST or upper critical solution temperature (UCST) polymers lead to nanocomposites that show thermally inducible flocculation behavior in the carrier medium [14–21]. The particles agglomerate at a critical temperature resulting in an enhanced magnetic response. Thus the agglomerated particles can be separated easily by low magnetic field gradients, and facilitate, for instance, the separation process in purification applications of biomolecules.

Reversible thermoflocculation of magnetic colloids by encapsulation with thermoresponsive polymers has been proposed based on thermoresponsive polymers such as poly(*N*-isopropylamide) (PNiPAAm) [15,21–24] and (oligoethylene glycol) methacrylate copolymers (POEGMA) [25], which both show an LCST type behavior, and on glycinamide copolymers with an UCST behavior at around 10 °C [20,26]. The formation of a polymer brush on the surface of single nanoparticles has proved to be a valuable tool for the design of single-cored hybrid structures with tailored dispersion behavior [17–19,21,27–30]. Magnetic polymer brushes with thermoflocculation behavior have been reported for organic solvents by our group [17–19]. Lately, hydrophilic brush shells have been described [31–34], prepared either by surface-initiated polymerization or by a “grafting to” approach of tailored copolymers from oligo(ethyleneglycol) methacrylates with adjustable and narrow flocculation temperature and low unspecific adsorption [25,35]. The ability of magnetic nanoparticles to show considerable heat dissipation due to relaxation processes is recently employed in the combination with thermoresponsive polymers [34,36–39]. In ac magnetic fields in the kHz range, the nanoparticles transform magnetic energy to heat energy due to relaxation processes and hysteresis losses [40–42].

Here, we report the investigation of a biocatalytically active carrier system that can be tuned by temperature or external magnetic fields. The hybrid nanostructures are obtained by the combination of magnetic nanoparticle cores with a thermoresponsive poly[oligo(ethylene glycol) methyl ether methacrylate] copolymer shell [17–18,33–34] and covalently attached protease trypsin as the biocatalytically active species. A reversible shell collapse at elevated temperatures is made responsible for significantly enhanced protease activity.

## Results and Discussion

The target of the present study is the development of magnetic nanocarriers with tunable enzymatic activity. For the realization of such a system, we use several components, each performing a specific function. The iron oxide magnetic core allows positioning and heat generation, owing to its behavior in static and dynamic magnetic fields. The polymer shell is the anchor for covalent attachment of the enzyme, and allows the introduction of thermoresponsive behavior. At the same time, it improves biocompatibility and stabilization against agglomeration. Finally, the immobilization of trypsin as a serine protease introduces biocatalytic activity. These components result in hybrid nanostructures, which serve as a recoverable reaction system that can be activated reversely by external ac magnetic fields, by using the thermal energy developed by magnetic heating of the superparamagnetic cores in combination with the thermoresponse of the shell.

**Scheme 1:**
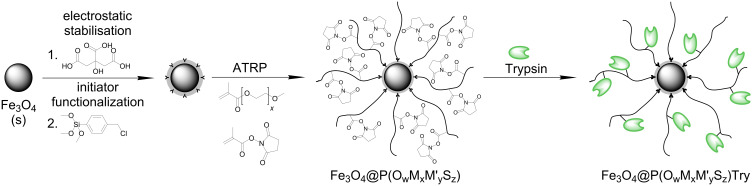
Synthesis of magnetic biocatalyst particles.

**Figure 1:**
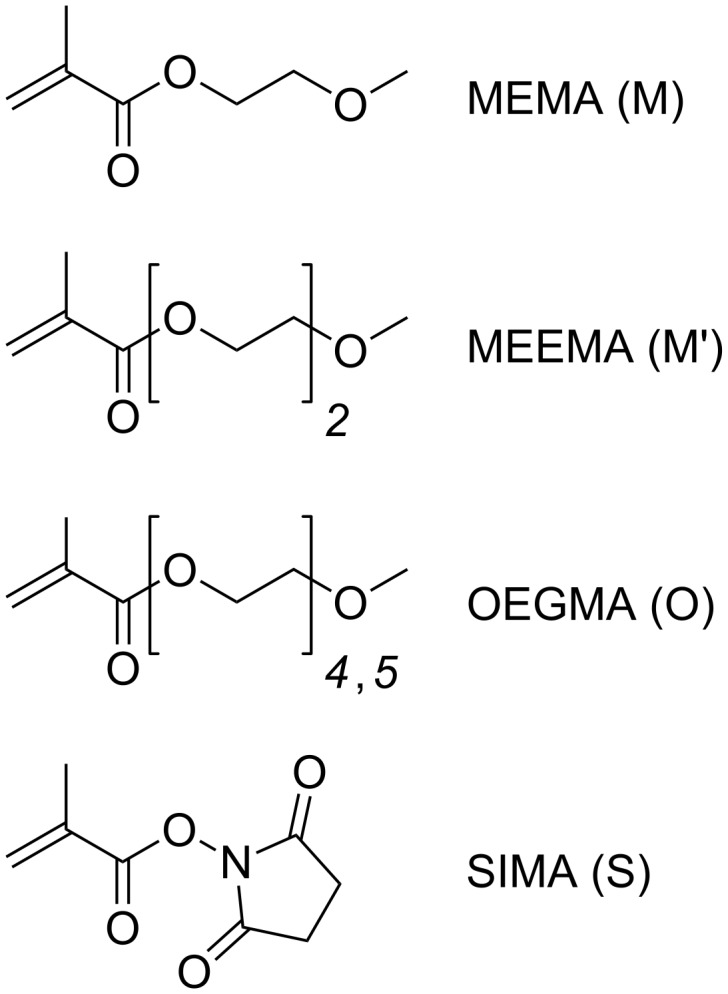
Structures of comonomers employed in the synthesis of functional core–shell particles.

These multifunctional hybrid particles are formed by surface initiated copolymerization of oligo(ethylene glycol) methyl ether methacrylates (labeled as MEMA (M), MEEMA (M’) and OEGMA (O)) with *N*-succinimidyl methacrylate (SIMA (S), Figure 1) as an active ester-functional monomer in DMSO by using surface-modified Fe_3_O_4_ nanoparticles as macroinitiators (Scheme 1). Subsequently, subunits carrying primary amine groups, such as proteins or enzymes, can be immobilized via the active ester pendant groups of the brush-type shell.

### Synthesis of functional core–shell nanostructures

In the first step, Fe_3_O_4_ nanoparticles are prepared by alkaline precipitation based on a method of Cabuil and Massart [43] and surface-functionalized with (*p*-chloromethyl)phenyltrimethoxysilane (CTS) [44] in order to introduce benzylic chlorine groups for subsequent initiation of atom transfer radical polymerization (ATRP). Oligo(ethylene glycol) methyl ether methacrylates with different length of the hydrophilic side chain are used as the main monomer to generate a hydrophilic polymer shell with tunable critical solution behavior in water. By proper choice of the copolymer composition, the thermoflocculation temperature of the core–shell particles can be adjusted [34–35]. The biocompatibility of poly(ethylene glycol) derivates is helpful to obtain nanoparticles acceptable for use in in vitro biological systems.

The direct introduction of carboxy functions to the polymer shell by surface-initiated ATRP involving (meth)acrylic acid is hindered by catalyst poisoning, resulting in a loss of reaction control. To overcome this, the protection of the carboxy group is useful [45], and in our approach, we employed succinimidyl methacrylate (SIMA) as a methacrylic acid derivative suitable for ATRP [46–48]. We have initially investigated the copolymerization behavior of the two monomers in model copolymerization experiments in solution, to ensure proper incorporation of the functional groups and stability of the active ester functionality under the polymerization conditions [33].

**Figure 2:**
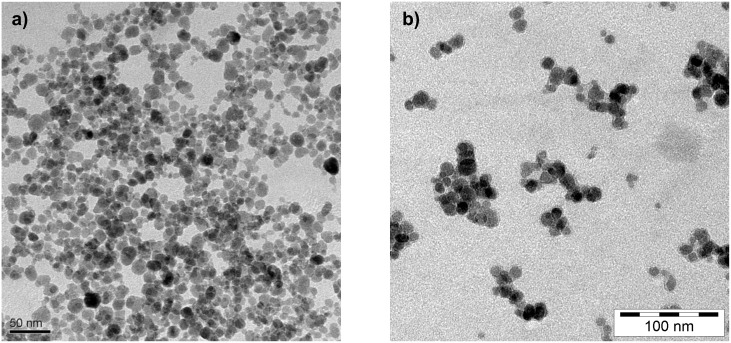
TEM images of a) Fe_3_O_4_ nanoparticles electrostatically stabilized by citric acid; b) Fe_3_O_4_@P(M_100_) nanoparticles after surface-initiated ATRP.

The catalytic system employed in the synthesis is based on copper(I) bromide and 2,2´-bipyridine in a DMSO solution. After 24 h of stirring at ambient temperature, viscous, deep brown magnetic dispersions are obtained. The success of the surface-initiated ATRP is qualitatively analyzed by transmission electron microscopy (TEM) and ATR-IR spectroscopy [33] on carefully washed and dried particles. TEM images (Figure 2) of the obtained nanoparticles demonstrate strongly contrasting Fe_3_O_4_ cores surrounded by less contrasting polymer shells. The nearly spherical nanoparticles are separately covered with a polymer layer of an average thickness of 3 nm, independent of the core size.

**Figure 3:**
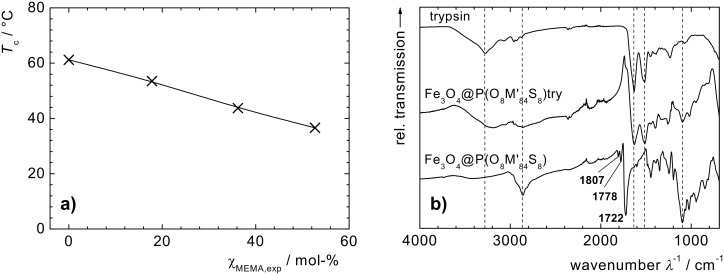
a) Cloud point temperature *T*_c_ of Fe_3_O_4_@P(O_x_M_y_) in water in relation to molar fraction of MEMA χ_M,exp._ in the polymer shell. b) ATR-IR spectra of trypsin, Fe_3_O_4_@P(O_8_M’_84_S_8_); and Fe_3_O_4_@P(O_8_M’_84_S_8_)-Try (dry powders).

ATR-IR spectra (Figure 3b) of the dry Fe_3_O_4_@P(O_x_M_y_S_z_) nanoparticles feature signals relating to the vibrational absorption of polymeric methyl and methylene groups (*ν* = 2800 cm^−1^–3050 cm^−1^), carbonyl double bond (*ν* = 1722 cm^−1^), C–O deformation (*ν* = 1099 cm^−1^) and N–O deformation (*ν* = 1025 cm^−1^), which clearly reveals the presence of P(OEGMA-*co*-SIMA). The three distinct peaks at *ν* = 1807 cm^−1^, 1778 cm^−1^ and 1722 cm^−1^ are characteristic of the vibrational absorption of the three carbonyl double bonds of the SIMA function, indicating that the succinimidyl ester is still existent and is neither hydrolysed nor deactivated [33]. The composition of the obtained hybrid materials is determined from mass loss between 150 °C and 480 °C in thermogravimetric analysis (TGA) (Table 1).

**Table 1:** Physical and chemical composition of investigated multifunctional core–shell nanoparticles.

Sample^a^	*μ*_P_ [wt %]	*d*_h,n_ [nm]	*d*_c_ [nm]	*μ*_MF_ [wt %]	*T*_c_ [°C]
					
Fe_3_O_4_@P(O_100_)	62.9	169	10.2	2.10	61.2
Fe_3_O_4_@P(O_82_M_18_)	43.8	127	10.7	1.91	53.4
Fe_3_O_4_@P(O_64_M_36_)	62.8	171	10.5	2.48	43.7
Fe_3_O_4_@P(O_47_M_53_)	33.8	113	10.3	1.22	36.6
Fe_3_O_4_@P(O_75_S_25_)	40.4	79	12.1	2.14	59.4
Fe_3_O_4_@P(O_80_S_20_)	49.9	47	10.3	1.54	57.8
Fe_3_O_4_@P(O_85_S_15_)	41.3	73	12.0	2.33	60.8
Fe_3_O_4_@P(O_90_S_10_)	52.1	49	10.4	1.51	57.9
Fe_3_O_4_@P(O_95_S_5_)	43.9	48	10.4	1.91	—
Fe_3_O_4_@P(O_8_M’_84_S_8_)	39.8	75	11.3	1.56	32.3

^a^Sample annotation: Fe_3_O_4_@P(O_w_M_x_M’_y_S_z_) with molar fraction of w: OEGMA, x: MEMA, y: MEEMA, and z: SIMA in the polymer shell; *μ*_P_: mass fraction of copolymer in dry particle powder (TGA), *d*_h,n_: number average hydrodynamic diameter (DLS), *d*_c_: volume average core diameter (VSM), *μ*_MF_: mass content of Fe_3_O_4_ in saturated DMSO dispersion (VSM), *T*_c_: cloud point temperature (CPP).

The hydrodynamic diameter of the core–shell nano-objects in aqueous dispersion can be detected by dynamic light scattering (DLS). A significant increase of the hydrodynamic diameter can be observed (Table 1), compared to electrostatic stabilized particles, and to CTS functionalized particles with a number average hydrodynamic diameter *d*_h,n_ of 14 nm and 21 nm, respectively.

### Hybrid particle characteristics

**Figure 4:**
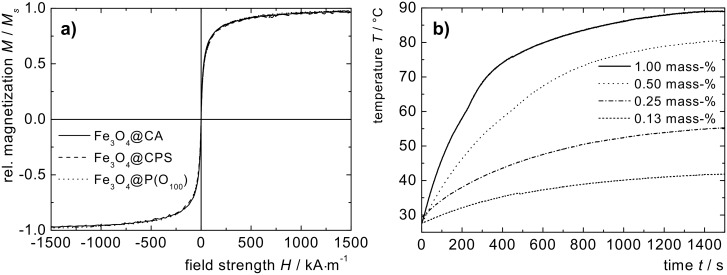
a) Normalized magnetization loops of dispersions based on Fe_3_O_4_@CA in water (solid line), Fe_3_O_4_@CPS in DMSO (dashed line), and Fe_3_O_4_@P(M_90_C_10_) in DMSO (dotted line); b) sample temperature *T* vs irradiation time *t* for Fe_3_O_4_@P(O_100_) nanoparticle dispersions in ac magnetic fields (250 kHz, H = 31.5 kA·m^−1^).

The magnetic properties of the nanoparticles at different points within the synthesis were investigated via vibrating sample magnetometry (VSM). In Figure 4a the magnetization curves of electrostatically stabilized nanoparticles Fe_3_O_4_@CA, initiator functionalized nanoparticles Fe_3_O_4_@CPS and magnetic polymer brushes Fe_3_O_4_@P(O_100_) are shown. Obviously, the graphs are almost perfectly matched after normalization by the saturation magnetization *M*_s_, demonstrating that the magnetization behavior of the magnetic cores does not change during the synthesis of the polymer brushes. Furthermore, it can be observed from the graphs that the particles’ cores are superparamagnetic and show no hysteresis in all investigated samples. By employing Chantrell´s method [49], we extract values for the core diameter between 10.2 nm and 10.7 nm from the initial slope (Table 1) [36].

As a consequence of the surface-initiated polymerization process, the polymer shells are end tethered to the particle surface in high density, as it has been previously shown by us and others [15,17]. When exposed to a suitable solvent, the polymer chains are highly solvated and protrude out from the surface in form of a polymer brush. In this state, the shell readily serves as a steric stabilizer for the particle dispersion, as the solvated brush surfaces result in a short-range repelling potential between individual particles. In contrast, when exposed to a bad solvent, the shell collapses, and particle agglomeration is observed.

In the present case, the polymer shell displays a reversible LCST behavior in water with a thermoflocculation temperature *T*_c_ that can be adjusted by the copolymer composition, as we have shown in a recent paper [34]. Thus, in aqueous media, the hybrid particles show thermoresponsive behavior [25]: while readily dispersible at low temperature, they reversibly flocculate when the dispersion temperature reaches the *T*_c_ of the shell, and therefore form a separate phase.

In the agglomerated state above the LCST, simple permanent magnets with magnetic field gradients below 50 mT·cm^−1^ are sufficient to separate the magnetic polymer brush particles from the carrier medium. We have shown that this behavior is of use for the easy magnetic separation of amino-functional probes and magnetically labeled biomolecules [33]. In this respect it is of interest to note, that the cloud point temperature can be adjusted by copolymerization in a wide range, including temperatures acceptable for biomolecules and biological species (Figure 3a) [34]. Furthermore, it has been shown that thermoflocculation of core–shell particles can be induced by magnetic heating of the particle cores in suitable ac magnetic fields [34,38].

The ability of superparamagnetic nanoparticles to locally develop heat when exposed to external ac magnetic fields in the kHz range is of considerable interest to activate physical or chemical processes in the vicinity of the particles, e.g., in hyperthermia [50–51], and for the remote operation of thermoresponsive soft actuators [39,52]. The heat development occurs due to relaxational processes (Néel and Brown) as well as hysteresis effects that results in considerable losses during the dynamic magnetic response of the materials [40,53–55].

We investigated the behavior of Fe_3_O_4_@P(O_100_) dispersions in an oscillating magnetic field (250 kHz, H = 31.5 kA·m^−1^) by recording the sample temperature with time (Figure 4b). The temperature of all samples increases within minutes to temperatures up to 80 °C depending on the FeO_x_ content. A higher Fe_3_O_4_ content leads to faster heating, and a specific heat power (SHP) = 86.5 W·g^−1^ of the particle cores can be extracted from the data. The generated heat flux is strong enough to reach the cloud point temperature *T*_c_ of 61 °C in the dispersions at magnetic fractions of *μ*(Fe_3_O_4_) = 0.5 mass % and higher. In this temperature range, we observe a slight deviation from the expected logarithmic deceleration of the heating rate (Figure 4b). We attribute the deviation to the heat consumption caused by the phase transition process.

### Enzyme immobilization and activity

**Scheme 2:**
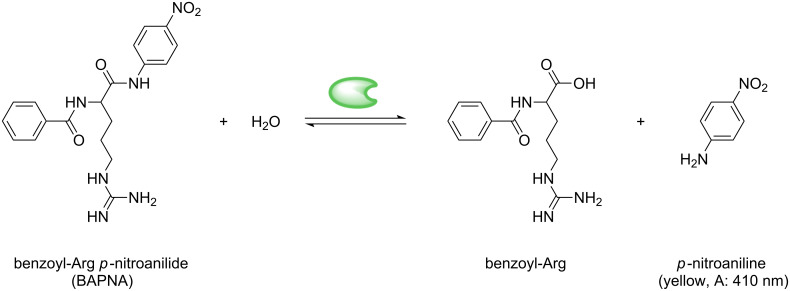
Reaction scheme of the enzymatic digestion of BAPNA catalyzed by magnetically labeled trypsin (
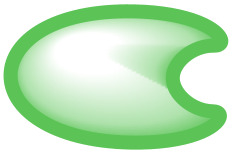
).

The immobilization of biomacromolecules on magnetic carriers is of interest for separable biocatalytic systems. We successfully immobilized trypsin as a model enzyme on the shell of Fe_3_O_4_@P(O_8_M´_84_S_8_) nanoparticles. Particles with low SIMA functionality have been chosen to avoid particle cross-linking or agglomeration due to multiple attachment of a single protein molecule by several nano-objects. Figure 3b compares the ATR-IR spectra of trypsin-functional nanoparticles (Fe_3_O_4_@P(M_8_O_84_S_8_)-Try) to free trypsin. In both samples, we observe similar amide signals (*ν* = 1620, 1578 cm^−1^), and also the NH-signal (*ν* = 3284 cm^−1^) of the trypsin peptide sequence is visible in both spectra. From the nitrogen content obtained by elemental analyses (EA), the amount of trypsin bound to the polymer surface of the nanoparticles is calculated to 111 mg trypsin per g particle (4.76 µmol·g^−1^), compared to commercially available magnetic particles for the protein binding with reported capacities between 1.5 mg·g^−1^ and 20 mg·g^−1^ [33,56]. The catalytic activity of trypsin, a protease for hydrolysis of specific peptide bonds (chain scission after the amino acids arginine and lysine), is investigated by the classical BAPNA method [57] and is compared to the native protein. Trypsin-catalyzed hydrolysis of benzoyl-Arg *p*-nitroanilide (BAPNA) results in the formation of *p*-nitroaniline that can be quantified by UV–vis spectroscopy at 410 nm (Scheme 2) [58].

The increase of relative absorption *A*_410_ is observed over time for different substrate concentrations *c*_BAPNA_. *A*_410_ is directly correlated to the *p*-nitroaniline concentration, thus the reaction rate *v* = d[*P*]/d*t* can be obtained from the initial slopes [2]. As a control experiment the primarily FeO_x_@P(O_8_M´_84_S_8_) nanoparticle dispersion without trypsin bound to the polymer shell is also used; no increase in UV absorption over time was detected in the control experiment. In every run employing either trypsin or immobilized trypsin, a linear increase of absorption with time can be detected in UV experiments for initial stages. To exclude possible trypsin leaching from the carriers, we continued the data collection for a couple of minutes after magnetic separation of the magnetic nanoparticles from the BAPNA solution. No further increase in adsorption was detected.

**Figure 5:**
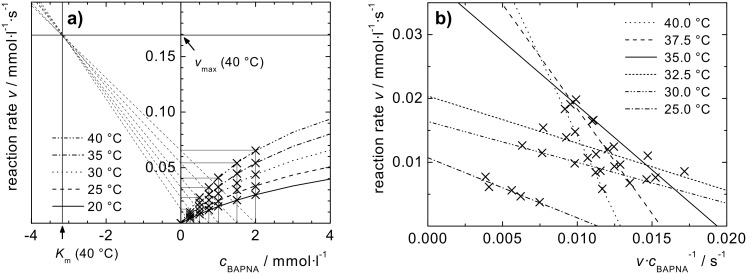
a) Cornish-Bowden diagram for the temperature-dependent kinetic data of trypsin activity, and b) Eadie–Hofstee diagram for the temperature-dependent kinetic data of nanocarrier activity.

By linearly plotting the reaction rates vs the BAPNA concentration *c*_BAPNA_ (Cornish-Bowden plot, Figure 5a), a hyperbolic behavior is observed that can nicely be fitted by the Michaelis Menten equation. The graph trends towards the saturation rate *v*_max_, and the Michaelis constant *K*_m_, respectively; where

[1]
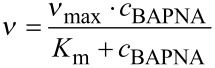


Data linearization can be achieved by the Eadie–Hofstee method [59] (Figure 5b) by plotting *v* against *v·c*_BAPNA_^−1^, and using the Michaelis–Menten equation in the form:

[2]
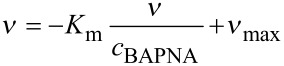


*K*_m_ and *v*_max_ can be determined from the negative slope and the intercept of the linear plots, respectively. A discontinuous behavior can be observed in the range of the LCST temperature (32.3 °C, see above) for aqueous Fe_3_O_4_@P(O_8_M’_84_S_8_)-Try dispersions.

**Scheme 3:**
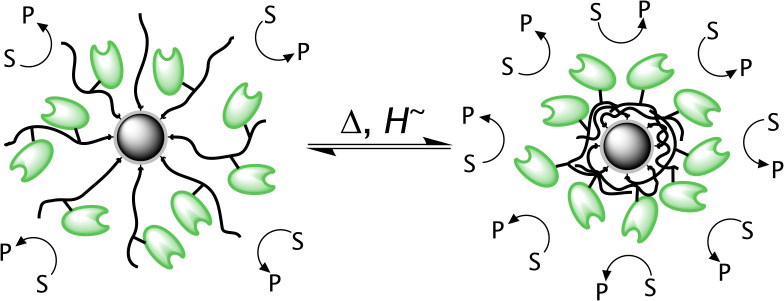
Proposed mechanism of catalytic activity after heating magnetic biocatalyst particles above *T*_c_.

**Figure 6:**
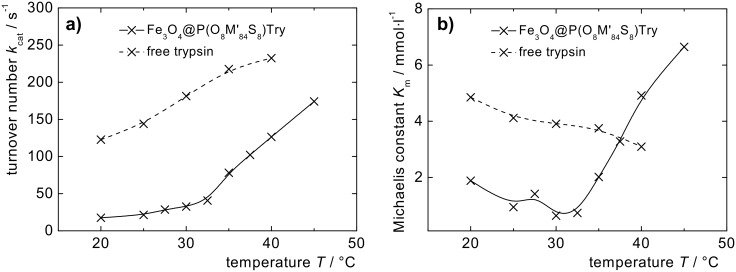
a) Turnover number *k*_cat_ and b) Michaelis constant *K*_m_ of particle- immobilized trypsin vs free trypsin.

While the Michaelis constant *K*_m_ of free trypsin decreases slowly with temperature, for particle-immobilized trypsin a strong increase is observed for temperatures above the *T*_c_ of the polymer, indicating a decrease in complex stability, probably due to shell collapse or particle precipitation, or both (Figure 6b). Nevertheless, the turnover number *k*_cat_ = *v*_max_/*c*_trypsin_ that is comparably low for particle-bound trypsin below *T*_c_, indicates a considerable acceleration of the particle-catalyzed reaction at temperatures above the *T*_c_ of the particle dispersion (Figure 6a). Probably, the shell collapse eases access to the enzyme for the substrate, considering that with a molar mass of 23,300 g·mol^−1^ the molecule size of trypsin is of the same order as *M*_n_ of the surface immobilized polymer chains (Scheme 3). In an upcoming study, the influence of diffusion and the extraction of thermodynamic parameters will give more insight to this process.

## Conclusion

In summary the presented results show that the combination of magnetic cores with a biocatalytically active, thermoresponsive polymer shell is a promising approach for nanoscopic carrier systems that allow an external reaction control and magnetic recovery of the catalyst. Fe_3_O_4_ nanoparticles with a brush shell composed of poly(oligo(ethylene glycol) methacrylates) and functionalized with porcine pancreas trypsin show temperature-tunable activity. Kinetic experiments on the catalytic activity by the BAPNA method support the reaction acceleration in particle-immobilized trypsin when the particles are heated across the transition temperature of the polymer shell, an effect that is attributed to the shell collapse. The particles can be heated by ac magnetic fields, resulting in remotely controlled biocatalytic systems. The principle described here is applicable to the modification of other biologically or catalytically relevant groups, and will therefore open new ways for the design of multifunctional hybrid nanostructures with different property portfolios.

## Experimental

**Materials:** Ammonium hydroxide aqueous solution (Fluka, 25%), *N*_α_-benzoyl-D,L-arginin-4-nitroanilide hydrochloride (BAPNA) (Sigma, 98%), benzylamine (BzA) (Janssen Chimica), 2,2´-bipyridine (bpy) (Aldrich, 99%), citric acid monohydrate (Grüssing GmbH, 99,5%), (4-(chloromethyl)phenyl)trimethoxysilane (CPS) (ABCR, 95%), copper(I) bromide (CuBr) (Aldrich, 98%), 1-(3-dimethylaminopropyl)-3-ethylcarbodiimide hydrochloride (EDC) (ABCR, 98%), *N*-hydroxy succinimide (NHS, Fluka), iron(III) chloride hexahydrate, iron(II) chloride tetrahydrate (Fluka, 98%), ninhydrin (Riedel-de-Haen), oligo(ethylene glycol) methylether methacrylate (OEGMA, Aldrich, *M*_n_ = 290 g·mol^−1^), 2-(2-methoxyethoxy)ethyl methacrylate (MEEMA), porcine pancreas trypsin type IX-S (Aldrich), tetramethylammonium hydroxide aqueous solution (25%) were used as received without further purification. Ethanol, diethyl ether and acetone were purified by distillation before use. Dimethyl sulfoxide (DMSO; min. 99.5%, Riedel-de-Haen) was distilled under reduced pressure from calcium hydride and stored under argon and molecular sieve (3A). HEPES buffer was prepared from 11 mM HEPES (Sigma), 140 mM NaCl (Merck), 4 mM KCl (Merck), 10 mM D(+)-glucose, and dissolved in deionized water. 2-Methoxyethyl methacrylate (MEMA, Aldrich, 99%) was distilled under reduced pressure and stored under argon. Nitric acid (conc., p.a., Merck) was diluted with distilled water resulting in a 2 N solution. Succinimidyl methacrylate (SIMA) was synthesized by a method by Gatz et al [26,60].

**Synthesis and stabilization of Fe_3_O_4_ nanoparticles:** The synthesis of magnetite nanoparticles on the gram scale was carried out by alkaline precipitation of iron(III) and iron(II) chloride following a method of Cabuil and Massart and is described in detail elsewhere [43]. For stabilization, the freshly synthesized nanoparticles were stirred with 420 mL 2 N nitric acid for 5 min. After washing with distilled water, 90 mL 0.01 N citric acid (CA) was added to the nanoparticles and stirred for 5 min. The particles were magnetically separated from the supernatant and 15 mL of tetramethyl ammonium hydroxide aqueous solution was added to obtain 3.32 g magnetic nanoparticles Fe_3_O_4_@CA in 92 mL of a stable dispersion at pH 8–9 (yield: 42.5%).

The Fe_3_O_4_ content *µ*(Fe_3_O_4_) in dispersion and the magnetic core diameter *d*_c_ were determined via VSM (*µ*(Fe_3_O_4_) = 2.55 mass%, *d*_c_ = 11.7 nm). DLS: *d*_h,n_ = 14.3 nm (25 °C in H_2_O). FT-IR (Diamond): *ν* (cm^−1^) = 2357, 2335 (C-N), 1247 (OH), 1098 (C-O), 1080 (OH).

**Surface modification of Fe_3_O_4_ nanoparticles:** For the immobilization of initiator sites on the particle surface of Fe_3_O_4_@CA, the dispersion was diluted with ethanol to a mass content of 1.0 g·l^−1^, and 1.80 mmol CPS per gram of Fe_3_O_4_ was added. After stirring for 24 h at ambient temperature, ethanol was removed under reduced pressure at 40 °C and the particles were washed with ethanol/acetone (1:1) five times. The particles were then redispersed in DMSO, resulting in a Fe_3_O_4_ content *µ*(Fe_3_O_4_) of 6.44 mass % (VSM) in dispersion (yield: 46.4%). The magnetic core diameter *d*_c_ was measured to be 11.1 nm (VSM). The functionalization degree of CPS was determined by EA to be 0.87 mmol CTS on 1.94 g Fe_3_O_4_@CPS. FT-IR (Diamond): *ν* (cm^−1^) = 2357, 2335 (C–N), 1241 (OH), 1115 (Si–O), 1011, 948 (Si–C).

**Surface-initiated ATRP of functional polymer shells:** The obtained CPS coated particles served as a macroinitiator for the following ATRP. The synthesis of Fe_3_O_4_@P(O_100_) is described, representatively. Therefore 6 mL of the DMSO-based particle dispersion (0.65 g Fe_3_O_4_@CPS) was mixed with 5 mL of a DMSO solution of 37.3 mg (0.26 mmol) CuBr and 101 mg (0.65 mmol) bpy. The polymerization was started by adding 5.83 mmol of the monomer (here: OEGMA). The mixture was stirred for 24 h at ambient temperature. The obtained viscous magnetic fluid was diluted with 10 ml DMSO to the final ferrofluid. The Fe_3_O_4_ content *µ*(Fe_3_O_4_) in dispersion and the magnetic core diameter *d*_c_ were determined via VSM. The polymer content *χ*_Pol_ in the dried particles was obtained from EA and TGA.

**Particle transfer to water/buffer:** The DMSO-based particle dispersion was added dropwise to diethyl ether (Et_2_O). The precipitate was washed five times with Et_2_O/Acetone (1:1) and was redispersed in distilled water or buffer to obtain an aqueous magnetic fluid.

**Immobilization of trypsin:** 30 mg trypsin was dissolved in 6 mL HEPES buffer and mixed with 6 mL of a HEPES buffer-based Fe_3_O_4_@P(O_85_S_15_) particle dispersion (*µ*(FeO_x_) = 0.15 mass %). In order to allow reactivation of active ester functions that may have hydrolyzed during storage, 6 mL of 2.21 μM EDC/NHS solution was added. The binding reaction was carried out for 6 h at ambient temperature on a shaker. The obtained trypsin functionalized particles were separated and washed carefully with water to remove any residues of free trypsin, and redispersed in HEPES buffer.

**Determination of immobilized enzyme kinetics and activity:** BAPNA was used as the model substrate. Four HEPES buffered BAPNA solutions with concentrations between 2.0 mM and 0.5 mM, and a 6.0 μM trypsin solution were prepared and tempered to the desired temperature. The respective BAPNA solution was added to a cuvette and mixed with 100 μL of FeO_x_@POEGMA-trypsin nanoparticle dispersion or with 50 μL trypsin solution. The cuvette was placed into the spectrophotometer and tempered. Starting with the addition of the enzyme, the change in absorption at 410 nm was detected over a period of up to 20 min by UV–vis spectroscopy.

**Analytic methods and instrumentation:** ATR-IR spectra were measured on a Nicolet 6700 spectrometer. Elemental analyses were performed on a Perkin-Elmer 2400 CHN analyzer. The organic content was calculated through C content. For TGA, a Netzsch STA 449c in a He atmosphere was used with a heating rate of 10 K·min^−1^ between 30 and 600 °C. Gel permeation chromatography (GPC) elugrams were collected on THF (300 × 8 mm^2^ MZ Gel Sdplus columns, Waters 410 RI-detector) relative to polystyrene standards. NMR spectroscopy was performed on a Bruker DRX500 at 500 MHz and ambient temperature. DLS experiments and zeta potential measurements were performed on a Malvern Zetasizer Nano ZS at 25 °C. The particle size distribution was derived from a deconvolution of the measured intensity autocorrelation function of the sample by the general purpose mode (non-negative least-squares) algorithm included in the DTS software. Each experiment was performed at least three times. Cloud point photometry of aqueous particle dispersions was performed on a Tepper TP1 cloud point photometer at 1 K·min^−1^ in HEPES buffer. From the turning point of the turbidity curves, the cloud point temperature *T*_c_ was obtained. Vibrating sample magnetization (VSM) measurements were implemented on an ADE Magnetics vibrating sample magnetometer EV7. Induction heating experiments were performed on a Hüttinger HF generator Axio 5/450T equipped with a copper inductor (l = 50 mm, dI = 35 mm, n = 5), and operating at 250 kHz and at a magnetic field of 31.5 kA·m^−1^. The experiments were performed in a vacuum-isolated glass sample container. Different samples with varying magnetite concentrations *μ*(Fe_3_O_4_) of Fe_3_O_4_@P(O_100_)-based magnetic fluid in water were exposed to the oscillating magnetic field. Via a fiber-optical sensor the fluid temperature *T* was measured against time *t*. For UV–vis spectroscopy, a Nicolet UV 540 spectroscope, a Unicam UV 500 or a Perkin Elmer Lambda19 with a thermostat Colora NBDS was used. Differential scanning calorimetry thermograms were collected on a Mettler-Toledo DSC 822^e^ at 5 K·min^−1^. TEM pictures were taken on a Hitachi H 600.

## References

[1] Tokarev I, Minko S (2009). Adv Mater.

[2] Keurentjes J T F, Kemmere M F, Bruinewoud H, Vertommen M A M E, Rovers S A, Hoogenboom R, Stemkens L F S, Péters F L A M A, Tielen N J C, van Asseldonk D T A (2009). Angew Chem, Int Ed.

[3] Kawaguchi H, Kisara K, Takahashi T, Achiha K, Yasui M, Fujimoto K (2000). Macromol Symp.

[4] Weissleder R (2001). Nat Biotechnol.

[5] Sun Y, Chen Z, Yang X, Huang P, Zhou X, Du X (2009). Nanotechnology.

[6] Pankhurst Q A, Thanh N K T, Jones S K, Dobson J (2009). J Phys D: Appl Phys.

[7] Misra R D K (2008). Mater Sci Technol.

[8] Horák D, Babič M, Macková H, Beneš M J (2007). J Sep Sci.

[9] Gu H, Xu K, Xu C, Xu B (2006). Chem Commun.

[10] Franzreb M, Siemann-Herzberg M, Hobley T J, Thomas O R T (2006). Appl Microbiol Biotechnol.

[11] Safarik I, Safarikova M (2004). Biomagn Res Technol.

[12] Pankhurst Q A, Connolly J, Jones S K, Dobson J (2003). J Phys D: Appl Phys.

[13] Safarik I, Safarikova M (1999). J Chromatogr, B: Biomed Sci Appl.

[14] Wakamatsu H, Yamamoto K, Nakao A, Aoyagi T (2006). J Magn Magn Mater.

[15] Sun Y, Ding X, Zheng Z, Cheng X, Hu X, Peng Y (2006). Chem Commun.

[16] Brazel C (2009). Pharm Res.

[17] Gelbrich T, Feyen M, Schmidt A M (2006). Macromolecules.

[18] Kaiser A, Gelbrich T, Schmidt A M (2006). J Phys: Condens Matter.

[19] Schmidt A M (2005). Macromol Rapid Commun.

[20] Ohnishi N, Furukawa H, Hideyuki H, Wang J, An C, Fukusaki E, Kataoka K, Ueno K, Kondo A (2006). NanoBiotechnology.

[21] Lai J J, Hoffman J M, Ebara M, Hoffman A S, Estournès C, Wattiaux A, Stayton P S (2007). Langmuir.

[22] Kondo A, Kamura H, Higashitani K (1994). Appl Microbiol Biotechnol.

[23] Heskins M, Guillet J E (1968). J Macromol Sci, Part A: Pure Appl Chem.

[24] Schild H G (1992). Prog Polym Sci.

[25] Chanana M, Jahn S, Georgieva R, Lutz J-F, Bäumler H, Wang D (2009). Chem Mater.

[26] (2010). Magnetic Beads Therma-Max.

[27] Perruchot C, Khan M A, Kamitsi A, Armes S P, von Werne T, Patten T E (2001). Langmuir.

[28] Chen X Y, Randall D P, Perruchot C, Watts J F, Patten T E, von Werne T, Armes S P (2003). J Colloid Interface Sci.

[29] Li D, Sheng X, Zhao B (2005). J Am Chem Soc.

[30] Li D, Jones G L, Dunlap J R, Hua F, Zhao B (2006). Langmuir.

[31] Wang S, Zhou Y, Guan W, Ding B (2008). Appl Surf Sci.

[32] Gou Z, Chen Y, Zhou W, Huang Z, Hu Y, Wan M, Bai F (2008). Mater Lett.

[33] Gelbrich T, Reinartz M, Schmidt A M (2010). Biomacromolecules.

[34] Gelbrich T, Marten G U, Schmidt A M (2010). Polymer.

[35] Lutz J-F, Stiller S, Hoth A, Kaufner L, Pison U, Cartier R (2006). Biomacromolecules.

[36] Kaiser A, Liu T, Richtering W, Schmidt A M (2009). Langmuir.

[37] Schmidt A M (2007). Colloid Polym Sci.

[38] Schmidt A M (2005). J Magn Magn Mater.

[39] Schmidt A M (2006). Macromol Rapid Commun.

[40] Hergt R, Dutz S, Müller R, Zeisberger M (2006). J Phys: Condens Matter.

[41] Müller G, Dutz S, Hergt R, Schmidt C, Steinmetz H, Zeisberger M, Gawalek W (2007). J Magn Magn Mater.

[42] Glöckel G, Hergt R, Zeisberger M, Dutz S, Nagel S, Weitschies W (2006). J Phys: Condens Matter.

[43] Massart R, Cabuil V J (1987). Chim Phys Phys-Chim Biol.

[44] Frickel N, Messing R, Gelbrich T, Schmidt A M (2010). Langmuir.

[45] Matyjaszewski K, Davis T P (2002). Handbook of Radical Polymerization.

[46] Rathforn J M, Tew G N (2008). Polymer.

[47] Shunmugam R, Tew G N (2005). J Polym Sci, Part A: Polym Chem.

[48] Monge S, Haddelton D M (2004). Eur Polym J.

[49] Chantrell R, Popplewell J, Charles S (1978). IEEE Trans Magn.

[50] Gneveckow U, Jordan A, Scholz R, Brüß V, Waldöfner N, Ricke J, Feussner A, Wust P, Hildebrandt B, Rau B (2004). Med Phys.

[51] Falk M H, Issels R D (2001). Int J Hyperthermia.

[52] Kaiser A, Winkler M, Krause S, Finkelmann H, Schmidt A M (2009). J Mater Chem.

[53] Néel L (1949). C R Hebd Seances Acad Sci.

[54] Brown W F (1959). J Appl Phys.

[55] Feyen M, Heim E, Ludwig F, Schmidt A M (2008). Chem Mater.

[R56] 56chemagen AG: activated M-PVA Magnetic Beads AK11, protein binding capacity 8–20 mg·g^−1^. http://www.chemagen.com/activated-m-pva-magnetic-beads.html (accessed June 1, 2010); micromod: nanomag®-D (streptavidin), streptavidin functionality 1.5–2.0 mg·g^−1^ (http://www.micromod.de/scripts/datasheet.asp?sid=929022981&prod=09-19-252&lng=g&typ=1, accessed June 1, 2010).

[57] Asgeirsson B, Cekan P (2006). FEBS Lett.

[58] Erlanger B F, Kokowsky N, Cohen W (1961). Arch Biochem Biophys.

[59] Lineweaver H, Burk D (1934). J Am Chem Soc.

[60] Batz H-G, Franzmann G, Ringsdorf H (1972). Angew Chem.

